# Supplementary material for: In situ reversible underwater superwetting transition by electrochemical atomic alternation
Source: Nat Commun. 2019 Mar 14;10:1212. doi: 10.1038/s41467-019-09201-1 (PMC6418196; doi:10.1038/s41467-019-09201-1)
Supplement: Supplementary file 1 — Supplementary information [file 41467_2019_9201_MOESM1_ESM.pdf]

In Situ Reversible Underwater Superwetting Transition by Electrochemical Atomic Alternation

Wang & Xu et al

Supplementary Information

Supplementary Note 1.

Theoretical analysis based on the minimization of the free energy for the whole underwater system. We introduce the theoretical model in terms of the minimization of a system's free energy to determine whether a solid will be wetted preferentially by water (E_w) or oil (E_o) underwater. The energetic gap between the two configurations is hence:

$$\Delta E = E_w - E_o$$

which can be further expressed as:

$$\Delta E = E_{\mathrm{w}} - E_o = R(\gamma_{ws} - \gamma_{os})S_{os} - \gamma_{ow}S_{ow}$$

According to the Young formula $\cos\theta = \frac{\gamma_{SV}-\gamma_{SL}}{\gamma}$, We thus have:

$$\Delta E = R\,(\gamma_{\mathrm{o}}\cos\theta_o - \gamma_w\cos\theta_w)\,S_{os} - \frac{\gamma_{\mathrm{o}}\cos\theta_o - \gamma_w\cos\theta_w}{\cos\theta_{ow}}S_{ow}$$

For the spherical cap with an angle of θ $(\theta < 10°)$:

$$S_{ow} = \frac{2}{1+\cos_\theta}S_{os} \approx S_{os}$$

Thus,

$$\Delta E = (R - \frac{1}{\cos\theta_{ow}})\,(\gamma_{\mathrm{o}}\cos\theta_o - \gamma_w\cos\theta_w)\,S_{os}$$

Where γ_O and γ_w are the surface tensions of the oil and the water, respectively, θ_{ow}, θ_o and θ_w are the equilibrium contact angles (CAs) of the oil in water, oil in gas and water in gas on a flat solid surface, respectively. R represents the roughness factor of the solid, which is defined as the ratio between the actual and projected areas of the surface. S_{os} is the apparent contact area of the oil-solid interface, and S_{ow} is the contact area of the oil-water interface.

When applying certain voltage, the solid is oleophilic underwater ($\theta_{OW} < 90°$), the condition $\gamma_{ow} = \frac{\gamma_o \cos\theta_o - \gamma_w \cos\theta_w}{\cos\theta_{OW}} > 0$ implies:

$$\gamma_o \cos\theta_o - \gamma_w \cos\theta_w > 0$$

Therefore, as long as $R - \frac{1}{\cos\theta_{OW}} > 0,$ we can have $\Delta E > 0$. That is to say, when the potential is on, the rough solid surface will be wetted preferentially by oil underwater.

When the potential is switched off, the solid is oleophobicity ($\theta_{OW} > 90°$) underwater where:

$$\gamma_o \cos\theta_o - \gamma_w \cos\theta_w < 0$$

$$R - \frac{1}{\cos\theta_{OW}} > 0$$

Therefore, we can have $\Delta E < 0$, which means the solid surface will be wetted preferentially by water underwater.

Supplementary Note 2.

Hybrid-energy-minimization (HEM). A hybrid-energy-minimization (HEM) technique (HyDro Droplet Simulator, AIST, H. Matsui, Japan) was used to evaluate the energy of droplets with different contact angles on solid surface. When a drop placed on a predefined homogeneous surface, the program will evolve droplet's shape gradually. So that as the steps increasing, the total energy decreased exponentially, and finally the droplet reaches to a minimum-energy state. Here, a 5 pL oil droplet was particularly used, and the calculation is stopped after the energy residual was estimated at <10^{-6} pJ. For superphobic to superphilic transition, the apparent CA of oil droplet changes from the value of > 150° at the initial contacting to an equilibrium state of < 15°. For superphilic to superphobic transition, the apparent CA changes from the value of < 15° at the initial contacting to an equilibrium state of > 150°.

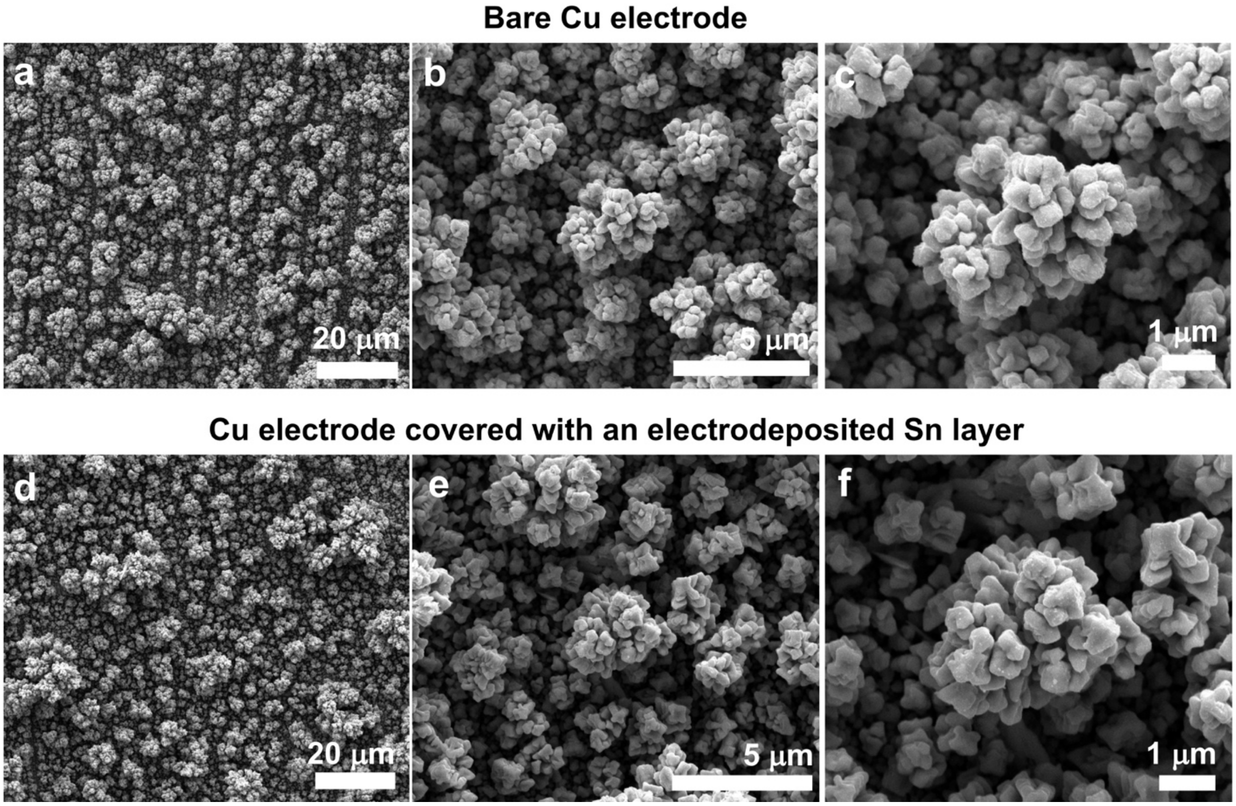

Supplementary Figure 1: SEM images of bare Cu electrode and the Cu electrode covered with an electrodeposited Sn layer. Both of them presented rough structures with micro- and nano-architecture features. To be noticed, here only very thin layer of Sn was deposited on the highly-textured Cu surface, by which surface energy changes drastically but the surface roughness almost remains.

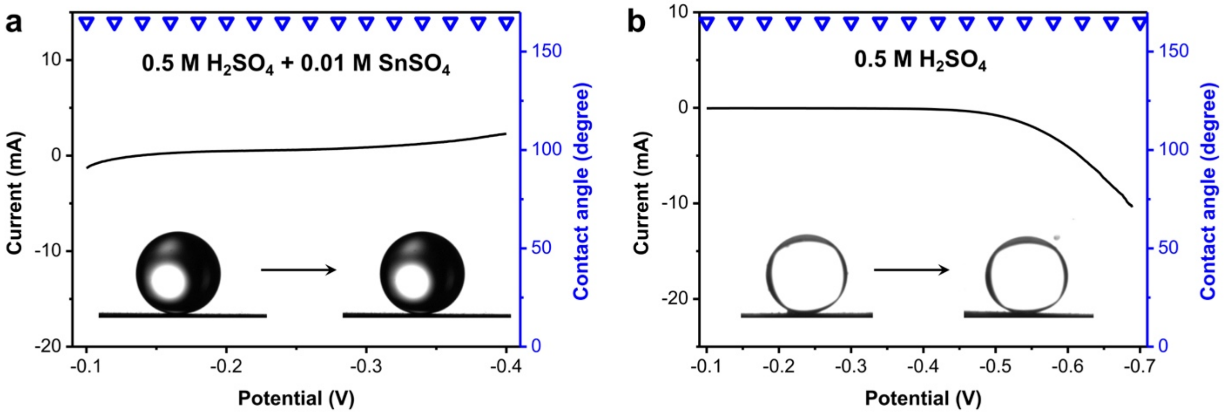

Supplementary Figure 2: The underwater superoleophobic state remained unchanged if (a) only UPD of Sn occurred in the potential region positive than -0.46 V for 0.5 M H_2SO_4 + 0.01 M $SnSO_4$ electrolyte, or (b) no Sn electrodepositing in the potential region negative than -0.46 V for 0.5 M H_2SO_4 electrolyte.

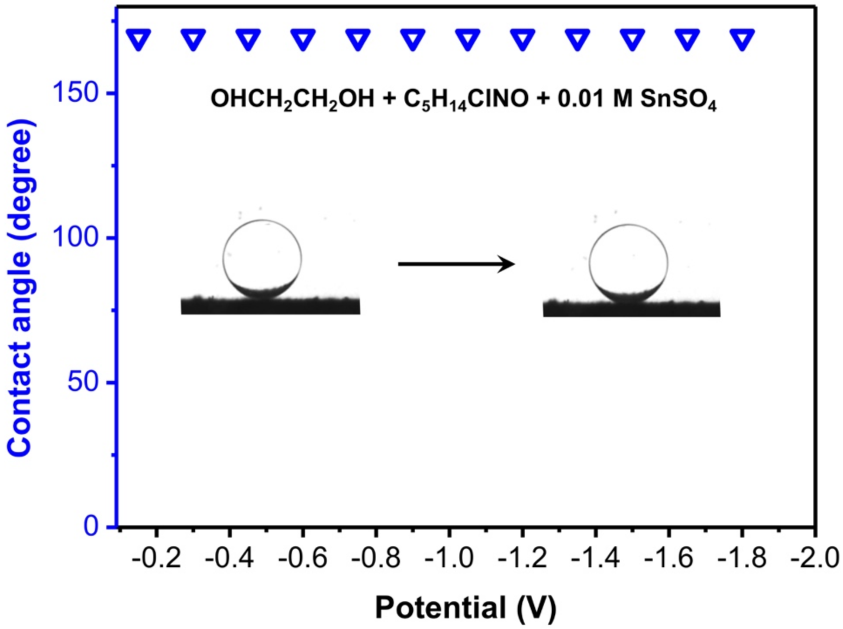

Supplementary Figure 3: No change for the surface wettability was observed when Sn was electrodeposited in a non-aqueous ionic liquid electrolyte, which demonstrated that the aqueous electrolyte was one of the main parameter that governs the *in situ* reversible underwater superwetting transition.

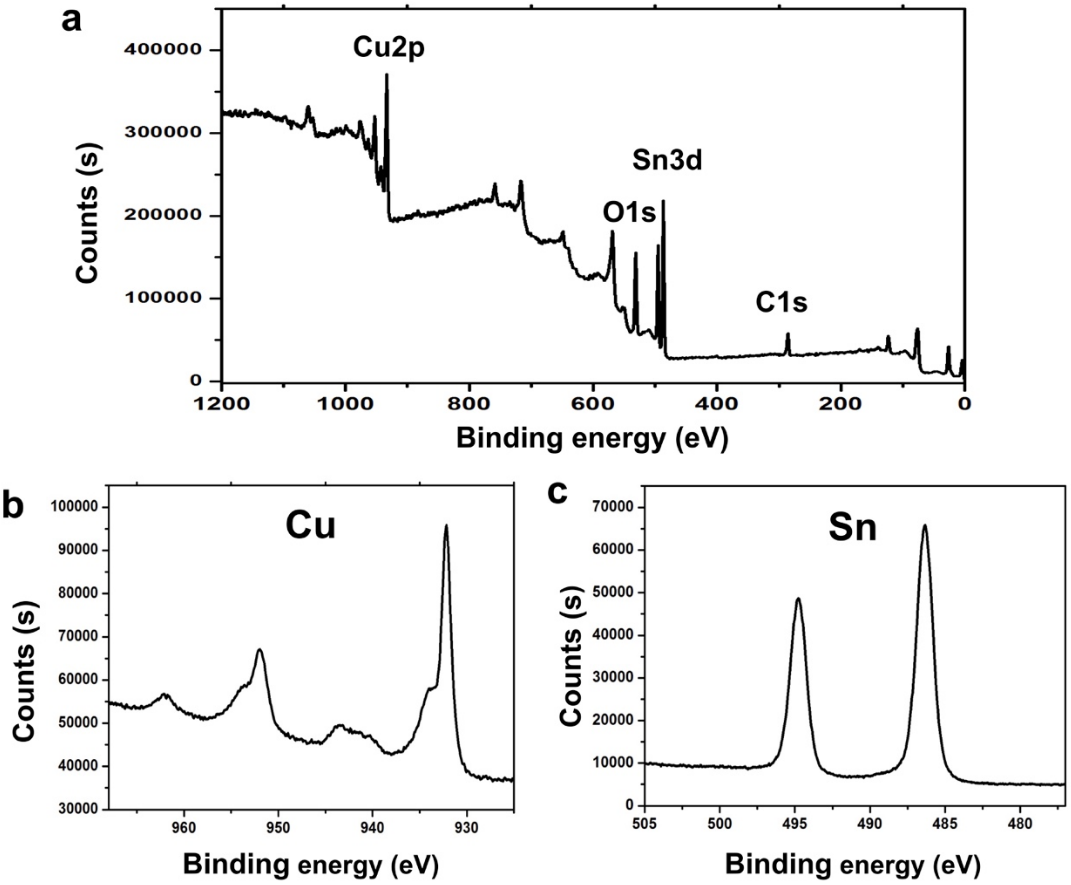

Supplementary Figure 4: XPS spectra shows that the surface was composed of the under-layer Cu electrode and the electrochemical deposited Sn element layer.

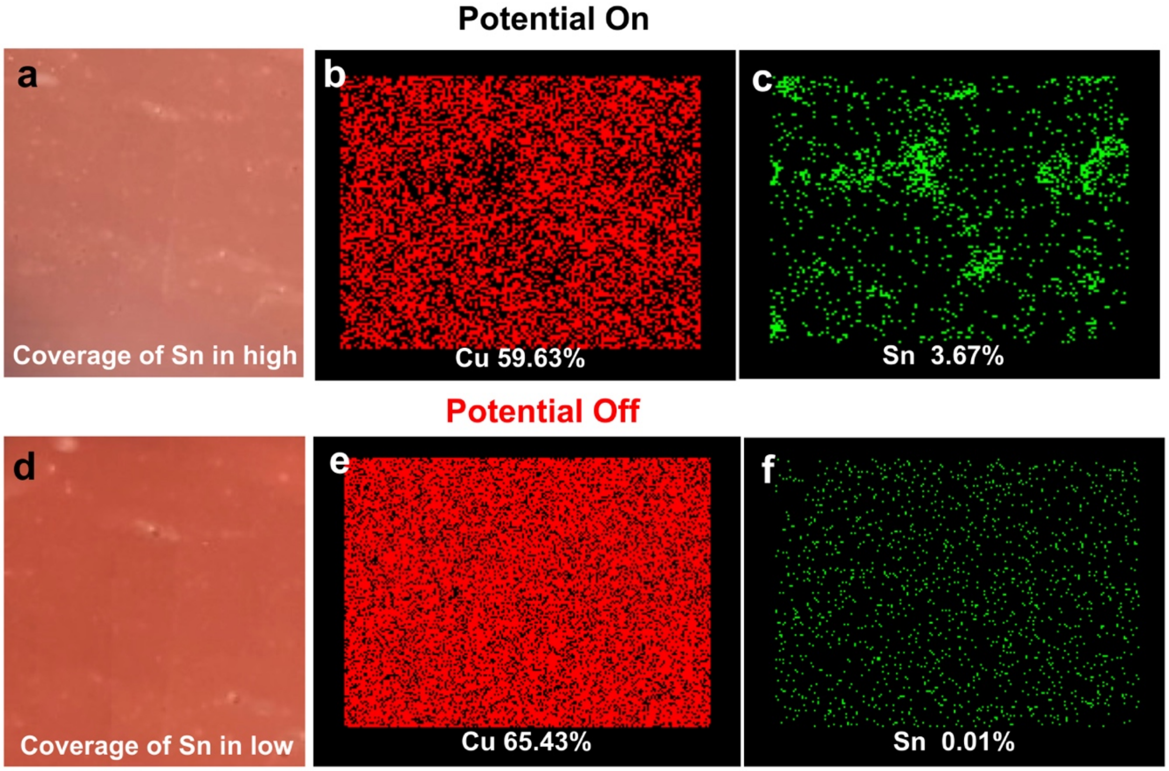

Supplementary Figure 5: The optical images of electrode and EDX mapping of Sn and Cu element on the Cu electrode with and without potential. (a-c) When the potential is on, the Cu electrode shows more bright red-brown in color, which is attributable to the abundance of Sn element on the surface. (d-f) When the potential is off, the Cu electrode shows dark red-brown in color, as a result of dissolving Sn into the electrolyte as Sn^{2+}.

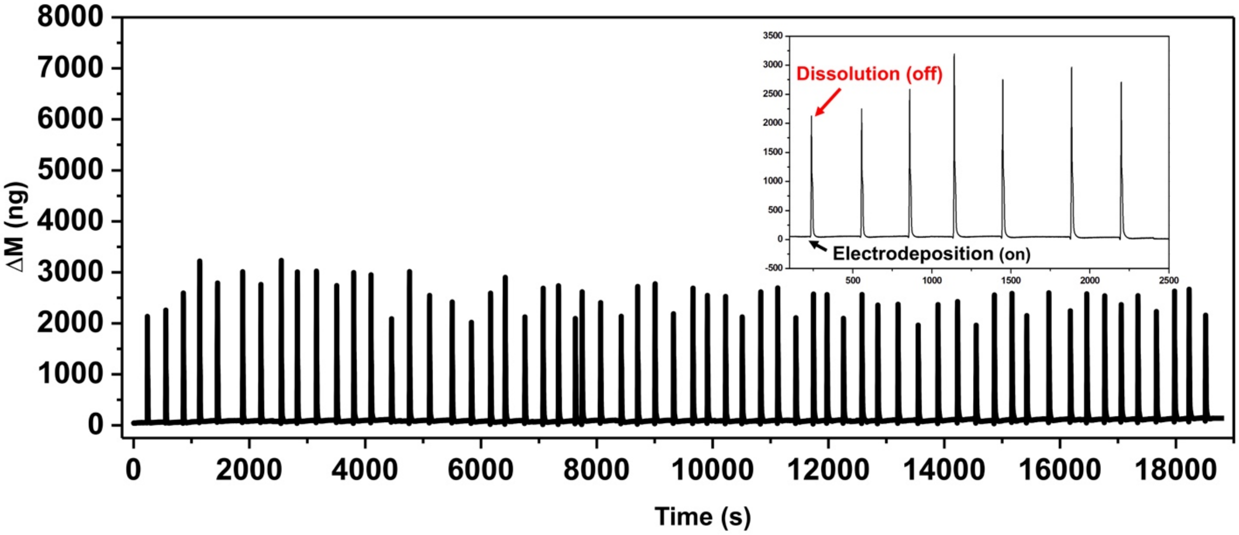

Supplementary Figure 6: Gravimetric measurements of Sn electrodeposited on a Cu electrode. It clearly showed that Sn presented a rapid increase in weight when applied potential, while, it would gradually dissolve into the electrolyte as Sn^{2+} when the potential was switched off. The deposition and dissolving of atomic Sn on the Cu electrode is reversible for multiple times with no clear accumulation of residual of Sn under experimental condition.

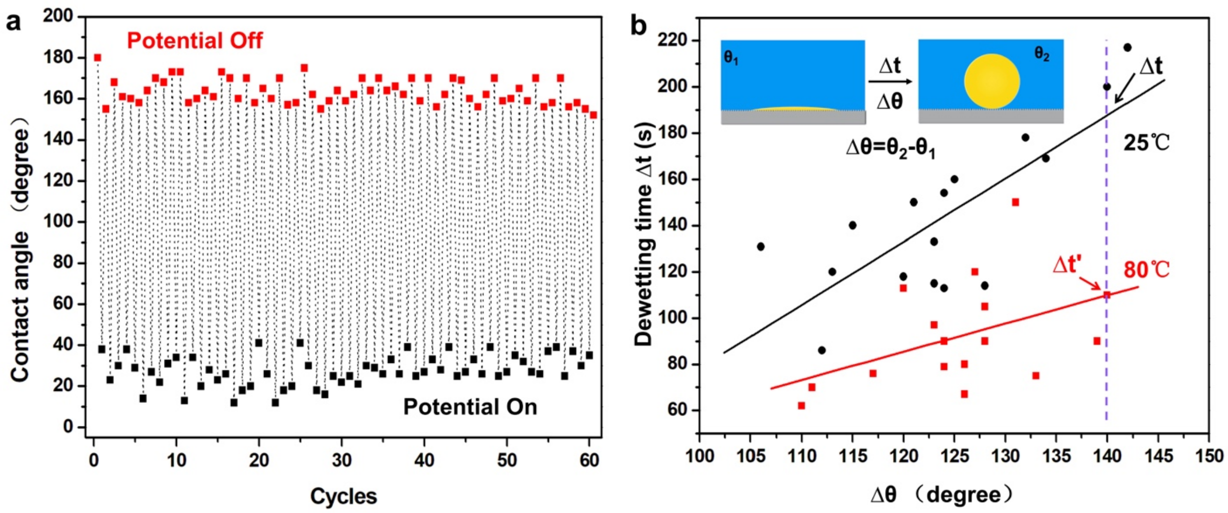

Supplementary Figure 7: (a) The reversibility of the *in situ* superwetting transition. It showed that the wettability transition of the electrode enjoy a good reversibility without observable decay of contact angle over 60 cycles. Specifically, in our system, the transition from a phobic to a philic state happens rather fast (within 1 s), while the inverse process takes comparative longer time ranging from ca. 80 to 200s depending on experimental condition. (b) The effect of temperature on the conversion time from a superphilic to a superphobic state with different variation range of CA (Δθ): the black curve indicates the wettability conversion at 25℃, and the red curve is the 80℃. The conversion time from superphilicity to superphobicity was largely shortened by increasing the temperature from 25℃ to 80℃, as a result of the enhanced Sn^{2+} diffusion.

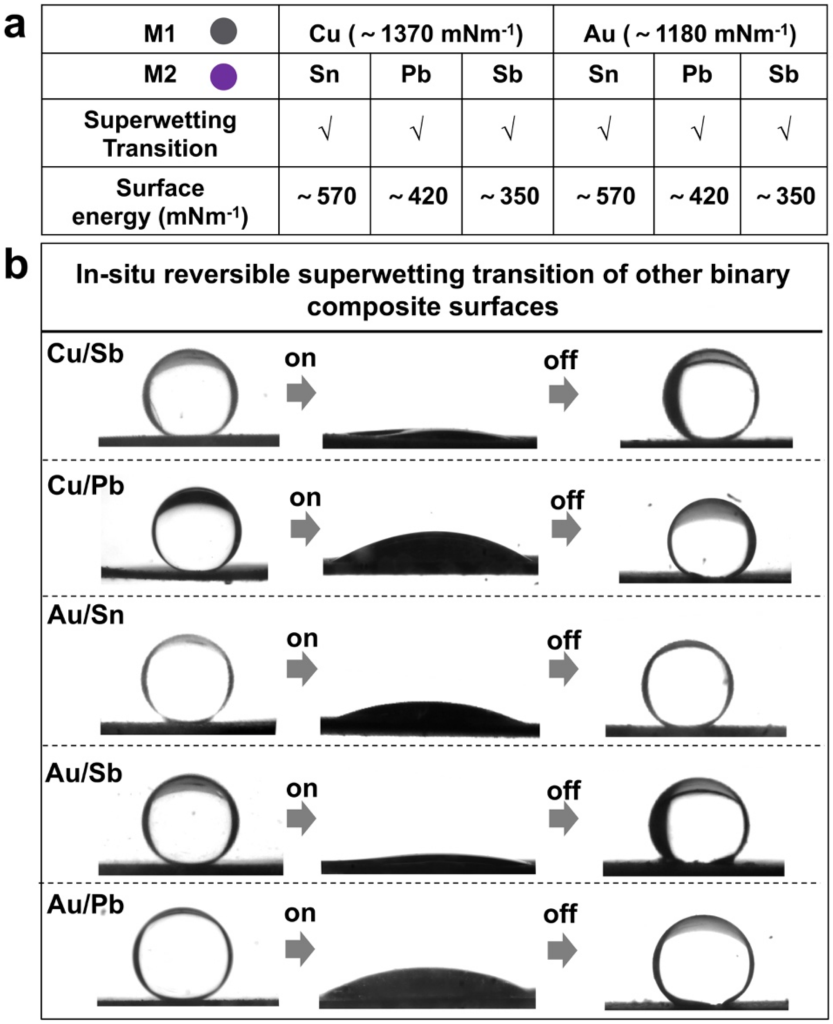

Supplementary Figure 8: (a) The concept of the metal-metal (M_1/M_2) binary composite surfaces that was composed of a high-surface-energy under-layer of M_1 atoms (e.g. Cu, Au) and a low-surface-energy deposited layer of M_2 atoms (e.g. Sn, Pb, Sb). (b) The *In-situ* reversible underwater superwetting transition of other binary composite surface, including Cu/Sb, Cu/Pb, Au/Sn, Au/Sb/ Au/Pb.

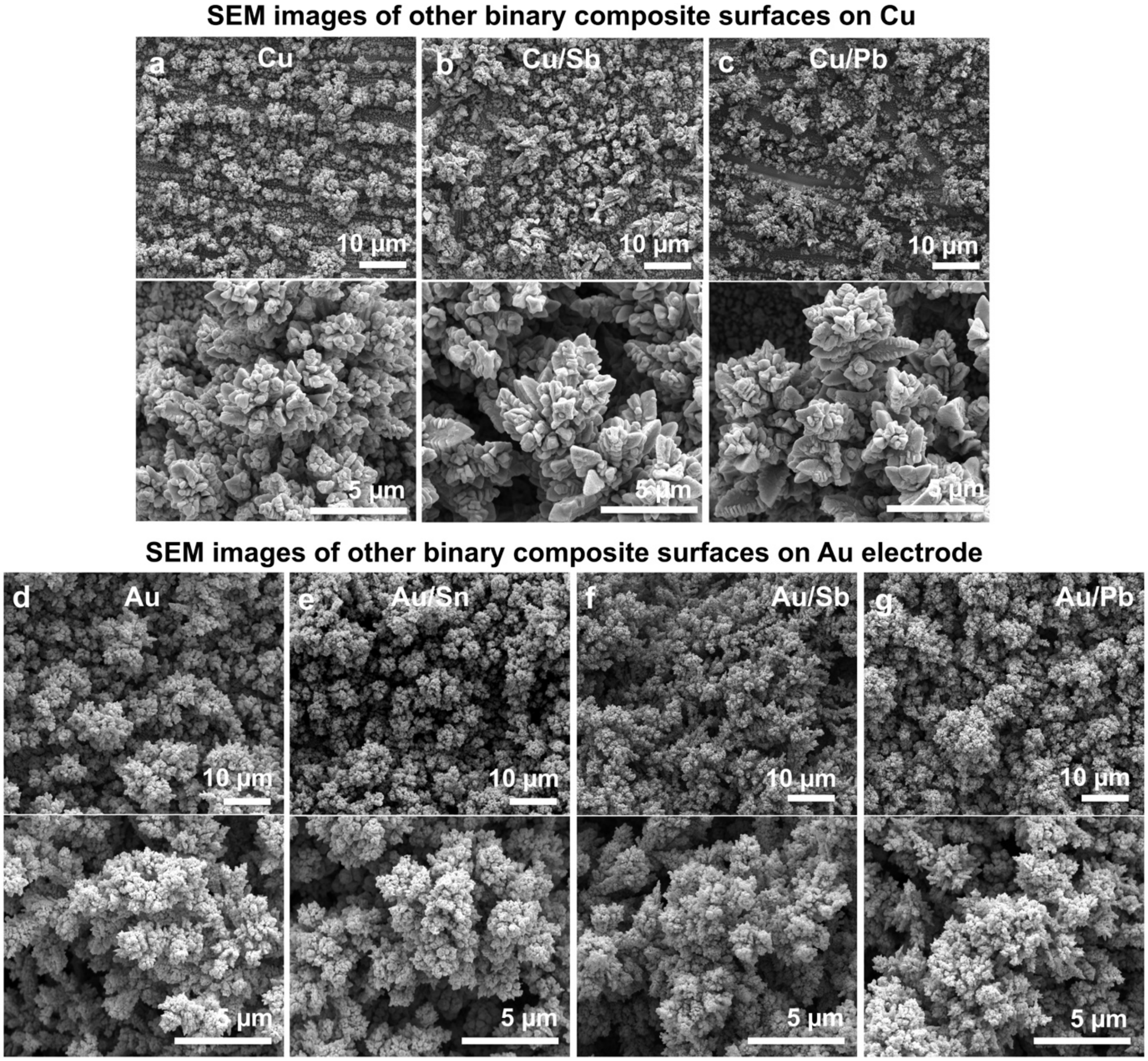

Supplementary Figure 9: (a) SEM images of bare Cu electrode and that covered with an electrodeposited foreigner atom of Sb and Pb layer, respectively, which is named as Cu/Sb, Cu/Pb binary composite surfaces. (b) SEM images of bare Au electrode and that covered with an electrodeposited foreigner atom of Sn, Sb and Pb layer, respectively, which is called as Au/Sn, Au/ Sb and Au/Pb binary composite surfaces. Both of them presented rough structures with micro- and nano-architecture features. To be noticed, here only very thin layer of foreigner atom was deposited on the highly-textured Cu (Au) surface, by which surface energy changes drastically but the surface roughness almost remains.

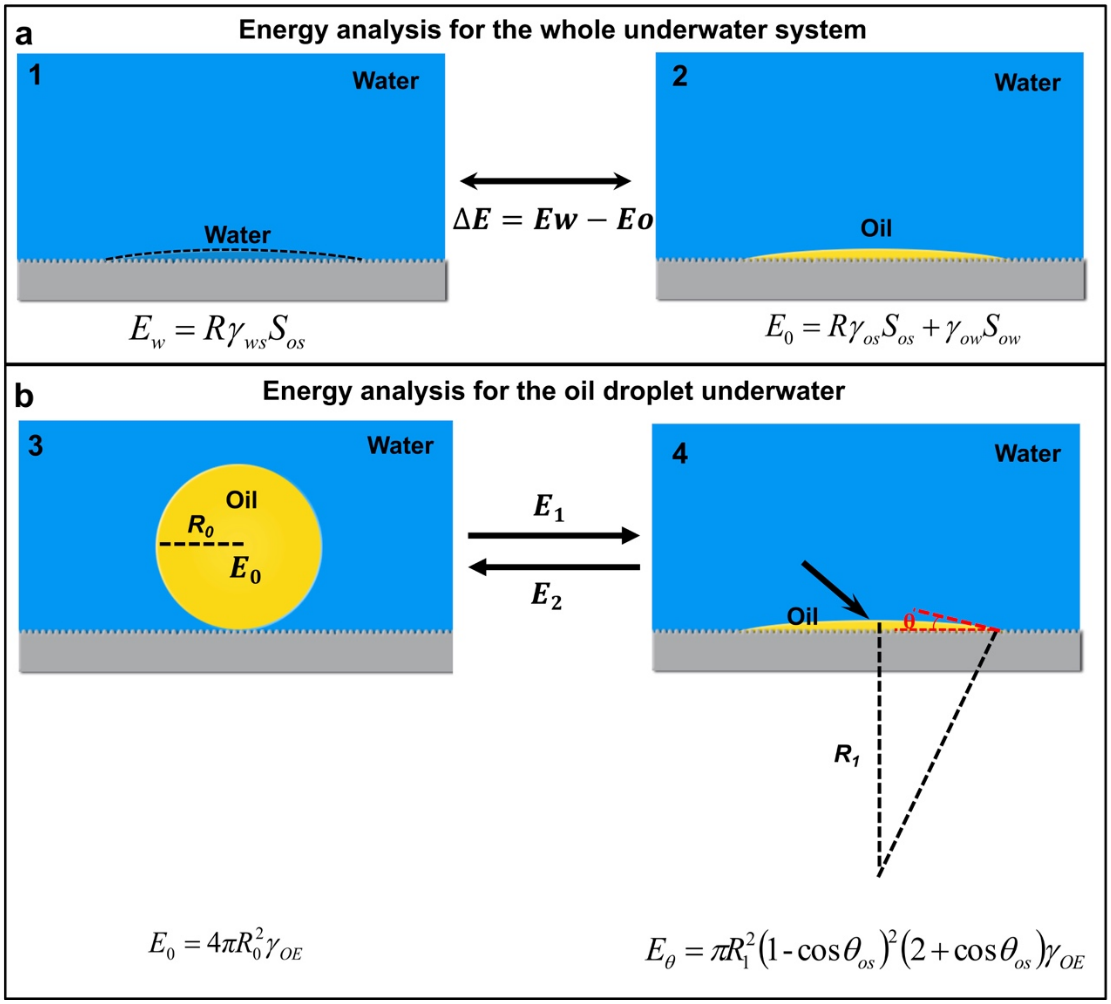

Supplementary Figure 10: Theoretical model based on the minimization free energy of the whole underwater system including the oil droplet. (a) The theoretical model in terms of the minimization of a system's free energy to determine whether a solid will be wetted preferentially by water (E_w) or oil (E_o) underwater. (b) The surface energy of the droplet with various CAs (E_θ) by theoretical calculation, where E_0 is the initial surface energy of droplet with CA of 180°, E_1 is the surface energy of droplet with CAs from ~ 180° to ~ 0° (from underwarer superoleophobicity to superoleophilicity), E_2 is surface energy of droplet with CAs of from ~ 0° to ~ 180° (from underwarer superoleophilicity to superoleophobicity).

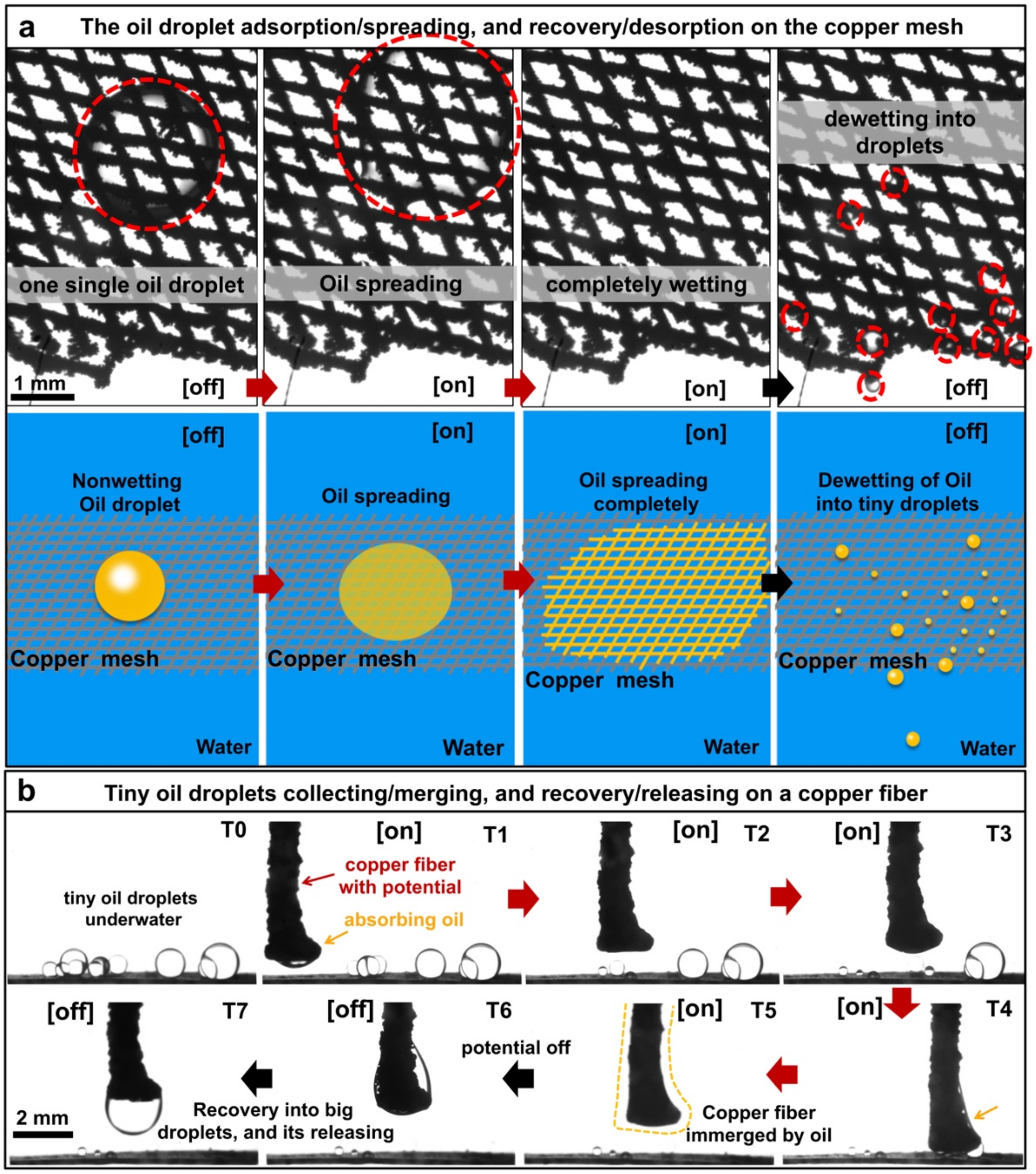

Supplementary Figure 11: Applications in oil collecting/recovery by a typical oil adsorption-desorption strategy. (a) a 5 μL oil droplet was adsorbed and totally spread on a copper mesh with a potential of -0.5 V. By switching off the potential, the completely adsorbed oil grandly retracted into numerous tiny droplets, and then drip off, finally, be released from the copper mesh automatically. (b) numerous tiny oil droplets were sequentially adsorbed and spread on a poised copper fiber, forming a continuous oil film on the copper fiber. After removing the potential, the continuous oil film retracted into a big droplet and released from the fiber.

Supplementary Movie 1.

Supplementary Movie 2.

Supplementary Movie 3.
