# Supplementary material for: In situ reversible underwater superwetting transition by electrochemical atomic alternation
Source: Nat Commun. 2019 Mar 14;10:1212. doi: 10.1038/s41467-019-09201-1 (PMC6418196; doi:10.1038/s41467-019-09201-1)
Supplement: Supplementary file 3 — Description of Additional Supplementary Files [file 41467_2019_9201_MOESM3_ESM.pdf]

Description of Additional Supplementary Files

File Name: Supplementary Movie 1

Description: In situ reversible transition between underwater superoleophilicity and superoleophobicity. When a 1,2-dichloroethaneoil droplet (2 μL) was released on a rough copper electrode under aqueous electrolyte without applying a voltage, it presented a perfect spherical shape with ~180o contact angle. Then a -0.5 V voltage was applied, the CA of oil droplet declined quickly from ~180o (underwater superoleophobic state) to ~10o (superoleophilic state) within less than 1 s. After removing the voltage, the spread oil film would gradually retract, and finally return to its original spherical shape.

File Name: Supplementary Movie 2

Description: The "adsorption-desorption" strategy in oil recovery application on a copper mesh. When a 5 μL oil droplet was carefully placed on a copper mesh, it remains perfect spherical shape due to the high-surface energy of the copper, namely, stays in a underwater superoleophobic state. Upon apply a voltage of -0.5 V, the oil droplet was adsorbed and totally spread on the copper mesh, showing typical underwater super superoleophilicity. By removing the voltage, the completely spread oil gradually retracted into numerous tiny droplets, which were released off from the copper mesh automatically.

File Name: Supplementary Movie 3

Description: The "adsorption-desorption" strategy in oil recovery application on a copper fiber. Using a copper fiber with certain potential, numerous tiny oil droplets were sequentially absorbed and spread on the copper fiber in a controllable manner, forming a continuous oil film on the copper fiber. After removing the potential, the continuous oil film retracted into a big droplet gradually and then released off from the fiber.
